# Detection of the antibiotic resistance genes content of intestinal *Bacteroides*, *Parabacteroides* and *Phocaeicola* isolates from healthy and carbapenem-treated patients from European countries

**DOI:** 10.1186/s12866-024-03354-w

**Published:** 2024-06-08

**Authors:** József Sóki, Ingrid Wybo, Zain Baaity, Glória Stefán, Samo Jeverica, Nurver Ulger, Catalina-Suzana Stingu, Bakhtiyar Mahmood, Katalin Burián, Elisabeth Nagy

**Affiliations:** 1 Institute of Medical Microbiology, Albert Szent-Györgyi Health Centre and Medical School, University of Szeged, Szeged, Hungary; 2 Department of Microbiology and Infection Control, Universitair Ziekenhuis Brussel, Vrije Universiteit Brussel, Brussels, Belgium; 3 National Laboratory of Health, Environment and Food, Maribor, Slovenia; 4 Department of Microbiology, Marmara University School of Medicine, Istanbul, Turkey; 5 Institute for Medical Microbiology and Virology, University Hospital of Leipzig, Leipzig, Germany; 6 Department of Biology, University of Garmian, Kalar, Kurdistan Region Iraq; 7 Department of Public Health, Government Office of the Capital City, Budapest, Hungary

**Keywords:** Antibiotic resistance, Antibiotic resistance genes, *Bacteroides*, *Parabacteroides*, *Phocaeicola*, Gut lumen, Gut mucosa, Intestinal microbiota

## Abstract

**Background:**

*Bacteroides fragilis* group (BFG) species are the most significant anaerobic pathogens and are also the most antibiotic-resistant anaerobic species. Therefore, surveying their antimicrobial resistance levels and investigating their antibiotic resistance mechanisms is recommended. Since their infections are endogenous and they are important constituents of the intestinal microbiota, the properties of the intestinal strains are also important to follow. The aim of this study was to investigate the main antibiotic gene content of microbiota isolates from healthy people and compare them with the gene carriage of strains isolated from infections.

**Results:**

We detected 13, mainly antibiotic resistance determinants of 184 intestinal BFG strains that were isolated in 5 European countries (Belgium, Germany, Hungary, Slovenia and Turkey) and compared these with values obtained earlier for European clinical strains. Differences were found between the values of this study and an earlier one for antibiotic resistance genes that are considered to be mobile, with higher degrees for *cfxA*, *erm*(F) and *tet*(Q) and with lower degrees for *msrSA*, *erm*(B) and *erm*(G). In addition, a different gene prevalence was found depending on the taxonomical groups, e.g., *B. fragilis* and NBFB. Some strains with both the *cepA* and *cfiA* β-lactamase genes were also detected, which is thought to be exceptional since until now, the *B. fragilis* genetic divisions were defined by the mutual exclusion of these two genes.

**Conclusions:**

Our study detected the prevalences of a series of antibiotic resistance genes in intestinal *Bacteroides* strains which is a novelty. In addition, based on the current and some previous data we hypothesized that prevalence of some antibiotic resistance genes detected in the clinical and intestinal BFG strains were different, which could be accounted with the differential composition of the *Bacteroides* microbiota and/or the MGE mobilities at the luminal vs. mucosal sites of the intestine.

**Supplementary Information:**

The online version contains supplementary material available at 10.1186/s12866-024-03354-w.

## Background

The microorganisms belonging to the Bacteroidota phylum may give a major pool (10–50%) of the human intestinal microbiota, of which some from the Bacteroidales order have great physiological and pathogenic potential, namely, the species of the former *Bacteroides* genus. They are now classified as *Bacteroides*, *Parabacteroides* and *Phocaeicola*. These strictly anaerobic species are significant contributors to the normal physiology of the gut, and they are also opportunistic pathogens. They interact with the host and the microbial community in several ways: pathogen exclusion, immunological tolerance, intestinal and microbiota maturation, degradation of dietary fibres and other biochemical activities. As anaerobic pathogens, they cause life-threatening conditions such as abdominal abscesses, other soft-tissue infections and sepsis, and they are the most frequently isolated anaerobic species in the clinic [1]. In particular, they are the most antibiotic-resistant anaerobes in terms of resistance levels and the number of resistance mechanisms [2]. The most significant opportunistic pathogenic *Bacteroides* species is *B. fragilis,* which is relatively rare in the intestine (1–10% of the *Bacteroides* count) but more frequent in infections (50–70% of *Bacteroides* cases) due to its higher potential for virulence [1]. Their empirical therapy is based on resistance surveys, which were frequent for the USA [3] and Europe [4] and were conducted from time to time in several westernized countries [5–10]; the number of developing countries where such studies were conducted is high or at least increasing [11–13]. The general trend is that very high (> 70%) resistance rates were obtained for penicillins, cephalosporins and tetracyclines; for cephamycins, MLS_B_ drugs such as clindamycin, and for moxifloxacin and amoxicillin/clavulanic acid, intermediate serious resistances (10–50%) were detected, and carbapenems, 5-nitroimidazoles, tigecycline and sometimes piperacillin/tazobactam were very effective with low (< 10–5%) resistance rates [2, 4]. What we know about the antibiotic resistance mechanisms of these antibiotics is sufficient but still not complete, and it is best for the most effective carbapenems and 5-nitroimiodazoles. Approximately 10% of *B. fragilis* strains can harbour the metallo-β-lactamase/carbapenemase gene, *cfiA*, which should be activated by insertion sequence (IS) elements for high resistance levels (MICs > 16 μg/ml) to develop, where the prevalence of such resistant strains is < 1–5% [14]. The *cfiA*-positive *B. fragilis* strains sometimes (1–5%) also have a particular phenotype, so-called heteroresistance, where more resistant subpopulations are in the cultures of the actual strains. An investigation of the molecular background of this latter resistance mechanism has just commenced [15]. The *cfiA*-positive subpopulation of the *B. fragilis* isolates also had a distinct phylogeny, and with typing methods, two genetic divisions were observed. One is *cepA*-positive (*cepA* is a Class A penicillinase/cephalosporinase) and *cfiA*-negative (Division I), and the other is *cepA*-negative and *cfiA*-positive (Division II) [16]. No exceptions to this mutually exclusive distribution have been described yet. Regarding the carbapenem resistance of *Bacteroides* strains, another metallo-β-lactamase was described recently in *B. xylanisolvens* strains [17]. The best known 5-nitroimidazole resistance mechanism for *Bacteroides* spp. and other anaerobic bacteria is that it is mediated by the *nim* genes, and in this case, the expression of the resistance gene is also upregulated by IS elements, but doubts have emerged that it is a single-gene resistance mechanism [18]. In addition to resistance surveys, the association of antibiotic resistance genes with resistance phenotypes has also been investigated extensively. In these cases, the exact activation mechanisms were not clarified fully, but the role of IS elements could be expected or proven in some cases (e.g., IS*4351* for the MLS_B_*erm*(F) or IS*Bf8* for the cephamycin resistance *cfxA* genes) [14].

In conjunction with the 2010 European *Bacteroides* antibiotic resistance survey [4], we also detected and correlated the resistance levels of the strains with their antibiotic resistance gene content, which was the most extensive of its kind [19]. Following these investigations, we conducted a study to isolate and measure the antibiotic susceptibility of *B. fragilis* group strains from healthy people and those who were treated with carbapenems, with the intention of determining their antibiotic resistance gene content and deriving novel, relevant information from additional comparisons to obtain a more detailed picture.

## Results

Out of the 241 BFG isolates included in our previous two studies, we chose 184 random isolates to test for antibiotic resistance genes [20, 21]. The association of these genes with the isolation parameter is shown in Table S2, and the differences are listed and explained in the Supplementary material.

To achieve our main goal of detecting differences in gene carriage between gut microbiotas/faecal samples and strains from infections, we performed χ2 tests, and the results are shown in Table 1. Most of the calculations gave nonsignificant results, but there were some instances where we found significant differences. The highest levels of divergence were found for the *cfxA* and *erm*(F) genes, which were represented more in faecal samples. The *msrSA, erm*(B) and *erm*(G) genes were also more abundant in gut microbiota strains, albeit with lower levels of statistical significance. Among gut microbiota strains, the *cfxA* and *tetQ* genes were represented with a higher prevalence in NBFB species.

**Table 1:**
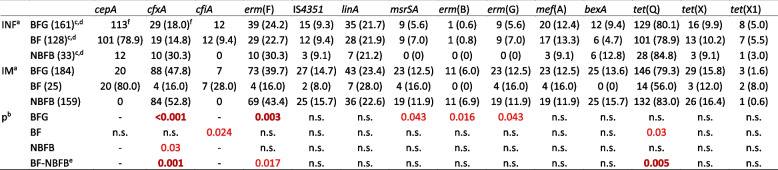
The differential distributions of antibiotic resistance genes

^a^INF and IM represent infections and intestinal microbiota, respectively; infection data were taken from our previous publication [19]. ^b^The significance values of differences; in bold and red for significance values <0.01, in red for significance values <0.05. ^c^Numbers of all the strains are in parentheses. ^d^BF stands for *B. fragilis*. ^e^Differences only between the intestinal microbiota BF and NBFB strains. ^f^The number of strains and their prevalence (in parentheses if meaningful) are shown in the given groups

**Fig. 1:**
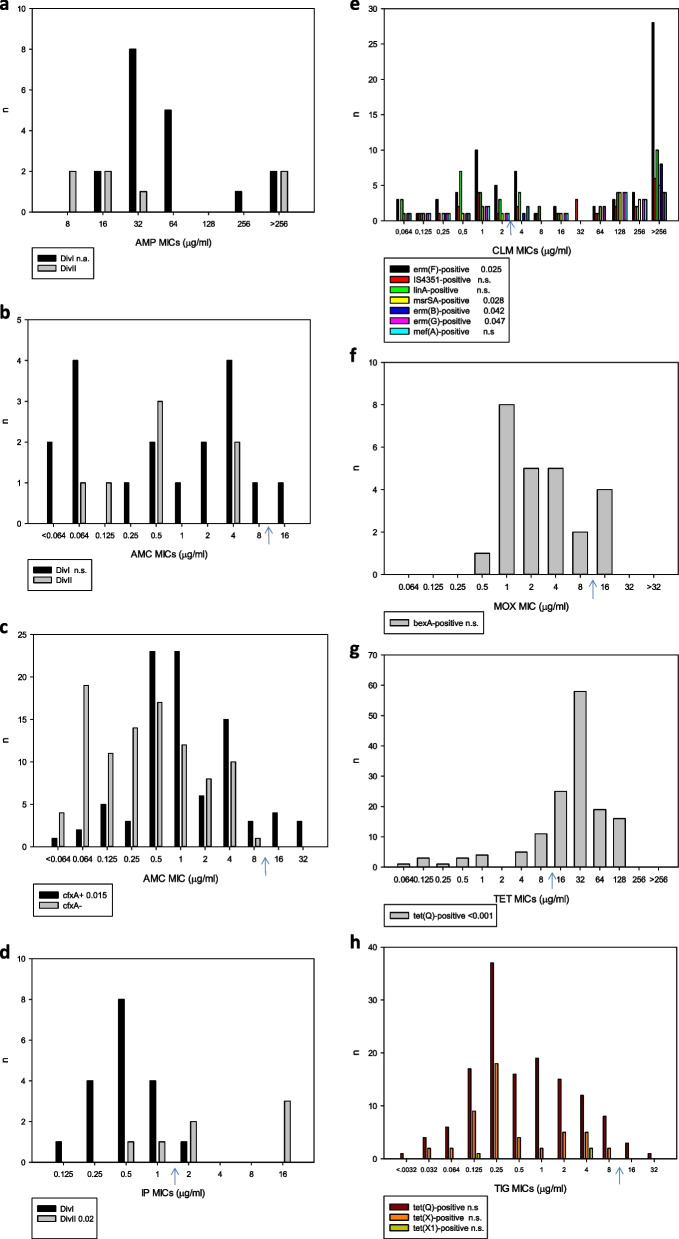
Distribution of the antibiotic resistance genes depending on the MICs of the target antibiotics. The significance values of χ2 tests that compare the resistance levels among strains with or without the actual antibiotic resistance genes or genetic divisions are shown in the legends after the genes. Here n.a. means not applicable. n.s. means not significant. These allow us to estimate the roles of the genes responsible for resistances to the antibiotics in question. Graphs a-g show the results for ampicillin, amoxicillin/clavulanic acid, cefoxitin, imipenem, clindamycin, moxifloxacin, tetracycline and tigecycline. The blue arrows denote the resistance breakpoints [22]. DivI and DivII denotes the two genetic divisions of *B. fragilis* (DivI—*cfiA*-negative, DivII—*cfiA*-positive). In case of ampicillin since the breakpoint is 4 μg/ml and the lowest value was 8 μg/ml blue arrow has not been shown

We also examined the relationship between the antibiotic resistance levels and the corresponding antibiotic resistance gene carriage (Fig. 1). Significant associations were found for the imipenem-*cfiA*, amoxicillin/clavulanic acid-*cfxA*, clindamycin-*erm*(F)*/msrSA/erm*(B)*/erm*(G) and tetracycline-*tet*(Q) antibiotic and resistance gene pairs.

Cross-correlation calculations also implied that there was a notable positive or negative association of antibiotic resistance gene carriages, which were in decreasing order as follows: *msrSA-erm*(G)-*mef*(A), *cfxA-erm(*F)*-tet*(X), *mef*(A)*-tet*(X1), *cepA-tet*(X1), *cepA-cfiA* (with correlation coefficients between approximately 0.2 and 0.9 of absolute values and with significances less than 0.01 down to approximately 10^–8^) and the *cepA-cfxA*, *cepA-tet*(Q) and *cfiA-cfxA* pairs with moderate negative correlations (Table 2). The low-level *cepA-cfiA* association was interesting because until now, it was thought that the carriage of these genes was 100% mutually exclusive in *B. fragilis* strains. In this case, this occurred because out of the 25 *B. fragilis* strains, 3 were double positive for these genes. Further examination showed that the detected *cepA* PCR products were deletion derivatives (312 bp, T_m_ = 80 °C) of the full-length *cepA* gene/PCR product (780 bp, T_m_ = 85 °C), and these 3 strains belonged to *cfiA*-positive genetic division II of *B. fragilis* detected by MALDI-TOF MS and ERIC12 PCR typing methods (data not shown).

**Table 2:**
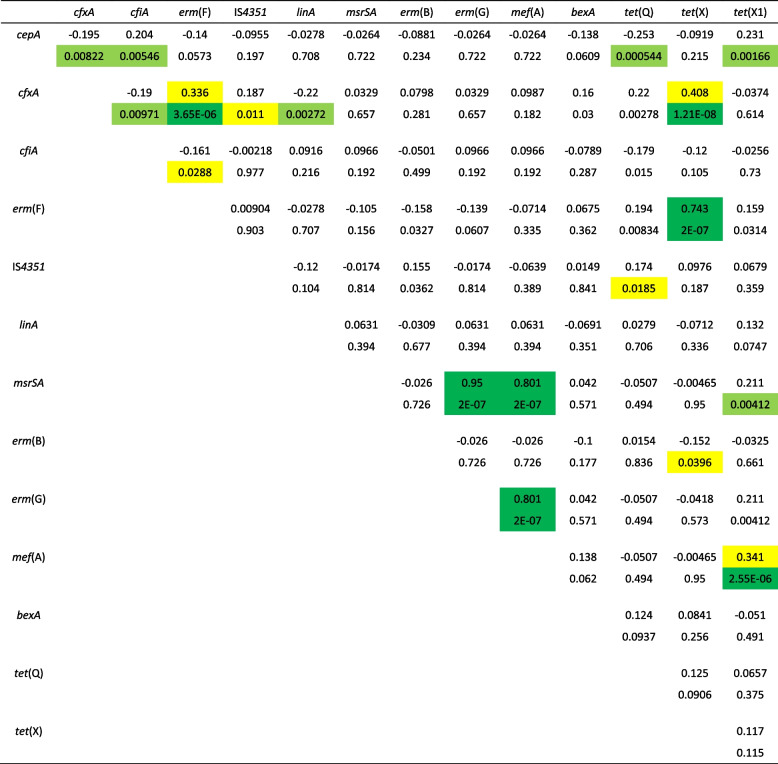
Cross-correlations of the presence of antibiotic resistance genes

Color-codings are as follows: yellow – 0.3<*r*<0.7. 0.05>*p*>0.01. light green – 0.01>*p*>0.00001. dark green – *r*>0.7. *p*<0.00001

## Discussion

BFG species, as members of Bacteroidetes, are important constituents of the intestinal microbiota and opportunistic pathogens, and their antibiotic resistance is also significant. Since they have the highest antibiotic resistance rates and numbers of antibiotic resistance mechanisms, they were anticipated as sources of antibiotic resistance genes in the complex ecosystem of the gut, as suggested by Salyers et al., mainly because they can harbour the *tet*(X) and *tet*(X1) aerobic tigecycline resistance genes on MGEs [23]. After screening the antibiotic resistance gene content of clinical BFG strains from Europe [19], we present a similar study on intestinal microbiota BFG strains in which we assessed the correlation of antibiotic resistance values with the corresponding resistance genes. We compared the results of the previous and current studies and examined the cross-correlations of the detected antibiotic resistance genes.

The roles of *Bacteroides* antibiotic resistance genes in antibiotic resistance were also confirmed in this study. As expected, the *cfxA* gene was involved in cefoxitin and β-lactam/β-lactamase inhibitor combination resistance, *cfiA* was the main determinant of carbapenem resistance among *B. fragilis* strains, *erm*(F), *erm*(B), *erm*(G) and *msrSA* were associated with clindamycin resistance, and *tet*(Q) was associated with tetracycline resistance. The β-lactamase inhibitor-resistant phenotype of one type of CfxA β-lactamase (the product of the proposed *cfxA1* gene) was described in the first publication that originally presented it [24].

Our comparisons of the prevalence of the antibiotic resistance genes between clinical and intestinal microbiota BFG strains demonstrated that some (*cfxA*, *erm*(F), *erm*(B), *erm*(G), *msrSA* and *tet*(Q)) are overrepresented in intestinal microbiota strains. Although the time dates of our two studies were different (2008–10 vs. 2014–16), the differences were in agreement with those in our previous publication, where an increase in the same antibiotic resistances was observed among Hungarian strains for the same period (2014–16) [22]. In these clinical studies with intestinal microbiota, there was an increase in resistance to cefoxitin, clindamycin, and tetracycline in the European strains with a concomitant decrease in resistance to amoxicillin/clavulanic acid and moxifloxacin. In Hungarian strains, cefoxitin and tetracycline resistance increased, and moxifloxacin resistance decreased, similar to that previously described [22]. For this study on the prevalence of antibiotic resistance genes, the same trend may apply, but other factors may be involved, namely, the different distribution of these two taxa (*B. fragilis* and NBFB) at these two anatomical sites (lumen and mucosa) [20]. (The effects of other isolation parameters could be mainly ruled out because of the balanced distributions, also see Table S2.) Therefore, for the prevalence of antibiotic resistance genes we suppose something similar as above. However, including the differential distribution of these two taxa (*B. fragilis* and NBFB) at these two anatomical sites (lumen and mucosa) might also cause these differences. Thus, we hypothesize the following explanations: (1) *B. fragilis* and the less virulent NBFB species have different distributions in the lumen and mucosa, and/or (2) HGT is more prevalent in the lumen. However, these two effects may be linked; the NBFB species are more abundant and have a higher HGT exchange in the lumen, whereas *B. fragilis* is mostly restricted to the mucosa and has lower HGT activities. To support our two related conjectures, we should mention the following findings. (1) It was found in several studies that the microbiota of the epithelial mucus layer and the lumen are different [25–27]. Furthermore, metagenomic sequencing studies demonstrated this difference for humans [28] and mice [29], and different microbiotas of the mucus layer and crypts were also detected in patients with colorectal cancers [30, 31]. *Akkermansia muciniphila* is a mucus-specific member of the gut microbiota [32], and the mucus layer and luminal microbiota compositions are regulated homeostatically [33]. The composition of the mucosal microbiota is regulated by biochemical activities such as inulin degradation [34], or it is affected by iron availability [35]. *B. fragilis* has mucus-degrading activity [36], and it was suggested that from the mucus layer, it can initiate pathological effects [37, 38]. (2) Highly significant (*p* < 0.001) differences were found in this study for *cfxA* and *tet*(Q) prevalence by variance analysis between *B. fragilis* and NBFB species (Table S2). Since the transfer of the MGEs of bacteria may be regulated by some antibiotics (the *tet*(Q) and *erm*(B) gene-carrying *Bacteroides* [39] and gram-positive bacteria [40], respectively), here we expect the same to apply to luminal and mucosal compounds/environments. Additionally, Coyne et al. described that *Bacteroides* strains are active in changing mostly ICEs in the faecal material of human individuals [41]. This supports our hypothesis that the differences in the antibiotic resistance gene contents of *Bacteroides* strains obtained from infections and from the intestinal microbiota taken from faeces were caused in a way described above.

Here, we also reported that some *cfiA*-positive faecal *B. fragilis* isolates were positive for a truncated version of the *cepA* gene. These isolates belonged to the *cfiA*-positive division II group of *B. fragilis*, as demonstrated by ERIC PCR and MALDI-TOF MS typing both have been proved to differentiate the two divisions of *B. fragilis* [16, 42]. Our study is the first to report such a co-occurrence, as all the examined *B. fragilis* strains to date carried exclusively the *cepA* or the *cfiA* gene. This dichotomy also means that the division I and II strains harbour some other differences in their genomes, and they can be easily typed by molecular methods. Recently, it was reported by others and by us that the *cepA* and *cfiA* genes and one other gene regulatory region are on specific chromosomal segments in the *B. fragilis* genomes that are at the same chromosomal loci and exclusively carry the distinguishing nucleotide sequences [43]. The determination of the full genomic sequence of one such ‘doubly positive’ *B. fragilis* strain (*B. fragilis* S82) is under way in our laboratory, and this may help us to fully elucidate its relation to the traits of the division I and II strains. Moreover, it would be interesting to assess the prevalence of such strains in the intestinal microbiota since we do not know if we found them by chance or if their prevalence is significant.

Among the other gene association/exclusion events, the cooccurrence of *erm*(G), *mef*(A) and *msrSA* and genes is readily explainable: they are usually harboured on the same genetic element, CTnGERM1 [44]. For the other detected association/exclusion relations, we do not have a precise explanation now, but molecular mechanisms responsible for their transfer and establishment in the cells should account for this.

## Conclusions

In this study, we investigated the prevalence of antibiotic resistance genes in intestinal microbiota BFG strains. A comparison with the gene content of BFG strains taken from infections revealed some increases, which we assume were caused by differential transmission at different anatomical sites of the intestine and by differential distribution in species of the BFG. Moreover, division II *B. fragilis* strains were also detected that harboured mutant *cepA* genes, which is a new finding. Our study encourages further investigations of the genetic transfer of antibiotic resistance determinants in the intestine, as it is a vital organ and a source of opportunistic infections.

## Methods

### Bacterial strains and cultivation

The bacterial strains used in this study were isolated from faecal samples of healthy people or from those of carbapenem-treated patient using BCA [45], an agar highly selective for *Bacteroides* sp., as described earlier [20, 21]. Briefly, one small loopful faecal material was suspended in 1 ml of BHI broth and then diluted 10^2^- and 10^4^-fold by sequentially adding 50 μL to 4950 µl of BHI broth and plating 100 μl aliquots on the surface of BCA plates with or without 4 mg/L meropenem [45]. The plates were incubated anaerobically at 37 °C for 48 h. Afterwards the approximate numbers of colonies grown were estimated and 3–8 colonies with different colony morphologies were picked and subcultured on SCA or on Columbia Blood agars and incubated with standard anaerobiosis (48 h) and aerobiosis (overnight), respectively. The species composition of the 184 test strains was as follows: *Bacteroides caccae* (9), *B. cellulosilyticus* (4), *B. clarus* (1), *B. eggerthii* (1), *B. finegoldii* (4), *B. fragilis* (25), *B. intestinalis* (3), *B. nordii* (2), *B. ovatus/xylanisolvens* (47), *B. stercoris* (4), *B. thetaiotaomicron* (24), *B. uniformis* (10), *Parabacteroides distasonis* (9), *P. johnsonii* (4), *P. merdae* (1), *Phocaeicola coprocola* (1) and *Ph. vulgatus/dorei* (35). The country and carbapenem treatment dependent species distributions are shown in Table S1. The isolates from carbapenem treated persons were also included since at the beginning we did not expect differences for which χ2 test test gave a final proof (Table S1). In Hungary and Belgium, the number of persons with and without carbapenem treatment were 4 + 11 and 4 + 4, respectively [21]. Regular cultivation of the isolates at Szeged, Hungary, was performed on Columbia agar supplemented with 5% sheep blood, 0.6 g/L cystein and 1 mg/L vitamin K_1_ (SCA) in an anaerobic cabinet (Concept 400, Ruskinn Technology Ltd., Bridgend, UK) with 70% N_2_, 10% H_2_ and 5% CO_2_ atmosphere as described earlier [20]. Bacterial strain identification was carried out by using MALDI-TOF MS (which cannot discriminate between the above species marked with a slash).

### RT-PCR, ERIC PCR typing and nucleotide sequencing

The detection of the antibiotic resistance genes *cepA*, *cfxA*, *cfiA*, *bexA*, *erm*(B), *erm*(F), *erm*(G) *linA*, *mef*(A), *msrSA*, *tet*(Q), *tet*(X) and *tet*(X1) and an IS element (IS*4351*) was carried out as described previously [19]. Briefly, Real-Time PCR reactions (10 μl) contained 5 μl mastermix (QuantiNova, Qiagen), 1 μl ROX reference dye 0.7 μM primers and 1 μl of template DNA prepared by the boiling method. Nucleotide sequencing of the *cepA* gene fragments of *cfiA*-positive strains were done by Sanger capillary sequencing outsourced to a provider (SEQOMICS Biotechnology Ltd., Hungary). ERIC PCR for strain typing which is able to make genomic fingerprints were also performed in the same way as described in our previous communication [42]. The GenBank submission number of the *cepA* gene sequence with a deletion is OQ718932.

### Statistical evaluation

For comparisons of the prevalence of the detected genes/elements, we used 1-way ANOVA and then Dunn’s method, Pearson and Spearman correlation counting, χ2 or Fisher’s exact tests by the Sigmaplot 12 software package. We also used this software to plot our figures.

### Supplementary Information

Supplementary Material 1.

## Data Availability

The entirety of the gene prevalence data generated and analysed during this study has been included in the published article. However, the raw data is available from the corresponding author upon formal request. The nucleotide sequence of the *cepA* gene in a *cfiA*-positive background is available in the GenBank database (https://www.ncbi.nlm.nih.gov/genbank/) under Acc. No. OQ718932.

## Abbreviations

BCA: *Bacteroides* Chromogenic agar
BFG: *B. fragilis* Group strains (formerly the *Bacteroides* species, which are now classified as *Bacteroides, Parabacteroides* and *Phocaeicola*)
ERIC: Enterobacterial repetitive intergenic consensus
ESCMID: European Society for Clinical Microbiology and Infectious Diseases
ESGAI: ESMID Study Group on Anaerobic Infections
HGT: Horizontal gene transfer
ICE: Integrative conjugative element
MALDI-TOF MS: Matrix-assisted laser desorption/ionization – time of flight mass spectrometry
MGE: Mobile genetic element
MLS_B_: Macrolide, lincosamide, streptogramin resistance phenotype B
NBFB: Non-*B. fragilis* BFG species
SCA: Supplemented Columbia agar
