# Supplementary material for: Detection of the antibiotic resistance genes content of intestinal Bacteroides, Parabacteroides and Phocaeicola isolates from healthy and carbapenem-treated patients from European countries
Source: BMC Microbiol. 2024 Jun 8;24:202. doi: 10.1186/s12866-024-03354-w (PMC11162026; doi:10.1186/s12866-024-03354-w)
Supplement: Supplementary file 1 — Supplementary Material 1. [file 12866_2024_3354_MOESM1_ESM.docx]

**Supplementary material**

**Table S1.** The composition of the test strains by species, sample type and origin of country

| Species | Hungary^a^ |  | Belgium |  | Germany | Slovenia | Turkey |
| --- | --- | --- | --- | --- | --- | --- | --- |
|  | n.t. | treated | n.t. | treated |  |  |  |
| *B. fragilis* | 14 | - | 2 | 1 | 3 | 2 | 3 |
| *B. ovatus/xylanisolvens* | 18 | 6 | 8 | 4 | 5 | 4 | 2 |
| *B. thetaiotaomicron* | 9 | 4 | 1 | 4 | 1 | 3 | 2 |
| *B. uniformis* | 2 | 1 | 1 | 3 | - | - | 3 |
| *P. vulgatus/dorei* | 8 | 5 | 5 | 4 | 6 | 3 | 4 |
| *P. distasonis* | 4 | 2 | 1 | - | - | 1 | 1 |
| other | 17 | 2 | 8 | 1 | 1 | 4 | 1 |

^a^ Number of strains, n.t. – not-treated with any carbapenem, for Germany Slovenia and Turkey only healthy people were involved.

χ^2^-tests did not detect any significant difference for the treated vs. non-treated groups or for all countries’ isolates.

**Table S2.** Variance analysis of the prevalence of antibiotic resistance genes depending on the isolation and taxonomic parameters

| Genes | MP Suppl^a^ | T-NT^a^ | Country | BF-NBFB^a^ | Species | DivI-II^a^ | |
| --- | --- | --- | --- | --- | --- | --- | --- |
| *cepA* | n.s.^b^ | n.s. | n.s. | <0.001 | <0.001 | | 0.005 |
| *cfxA* | n.s. | n.s. | <0.001 | <0.001 | 0.002 | | n.s. |
| *cfiA* | n.s. | n.s. | n.s. | <0.001 | n.s. | | <0.001 |
| *erm*(F) | 0.003 | n.s. | <0.001 | 0.009 | n.s. | | n.s. |
| IS*4351* | n.s. | n.s. | n.s. | n.s. | n.s. | | n.s. |
| *linA* | n.s. | n.s. | n.s. | n.s. | n.s. | | n.s. |
| *msrSA* | n.s. | n.s. | n.s. | n.s. | n.s. | | n.s. |
| *erm*(B) | n.s. | n.s. | n.s. | n.s. | n.s. | | n.s. |
| *erm*(G) | n.s. | n.s. | n.s. | n.s. | n.s. | | n.s. |
| *mef*(A) | n.s. | n.s. | n.s. | n.s. | n.s. | | n.s. |
| *bexA* | n.s. | n.s. | n.s. | n.s. | <0.001 | | n.s. |
| *tet*(Q) | n.s. | n.s. | n.s. | 0.002 | n.s. | | n.s. |
| *tet*(X) | n.s. | n.s. | <0.001 | n.s. | n.s. | | n.s. |
| *tet*(X1) | n.s. | n.s. | n.s. | n.s. | n.s. | | n.s. |

^a^ Table headings are as follows: MP Suppl – BCA with or without meropenem supplementation (4 μg/ml). T-NT – isolated from carbapenem-treated or non-treated patients. BF-NBFB – *B. fragilis* or non-*B. fragilis* *Bacteroides*. DivI-II – genetic division I or II of *B. fragilis*. ^b^ n.s. – non-significant or the significance values are shown.

The above results express whether there were significant differences between of the patients’ and taxonomic groups by the Dunn’s method. Interestingly. More *ermF* genes could be found among the strains isolated on meropenem-supplemented agars (which had slightly lower numbers that the non-supplemented ones) while the carbapenem treatment did not lead to elevations in the prevalence of any β-lactamase genes. Country-specific differences were found for the *cfxA* (Hungary vs. Slovenia). *ermF* (Turkey vs. Slovenia) and *tetX* (Hungary vs. Belgium, Germany and Slovenia) genes. The *B. fragilis*-specific *cepA* and *cfiA* genes did show differences in the taxonomic groupings and among the genetic divisions of *B. fragilis*. There were more *cfxA,* *ermF* and *tetQ* genes in non-fragilis *Bacteroides.* *P. distasonis* contained more *cfxA* than *B. fragilis* and *B. thetaiotaomicron* contained more *bexA* than *P. distasonis*.
